# Supplementary material for: Indol-3-Carbinol and Quercetin Ameliorate Chronic DSS-Induced Colitis in C57BL/6 Mice by AhR-Mediated Anti-Inflammatory Mechanisms
Source: Int J Environ Res Public Health. 2021 Feb 25;18(5):2262. doi: 10.3390/ijerph18052262 (PMC7956562; doi:10.3390/ijerph18052262)
Supplement: Supplementary file 1 [file ijerph-18-02262-s001.pdf]

Supplementary Materials:

The following are available online at www.mdpi.com/xxx/s1.

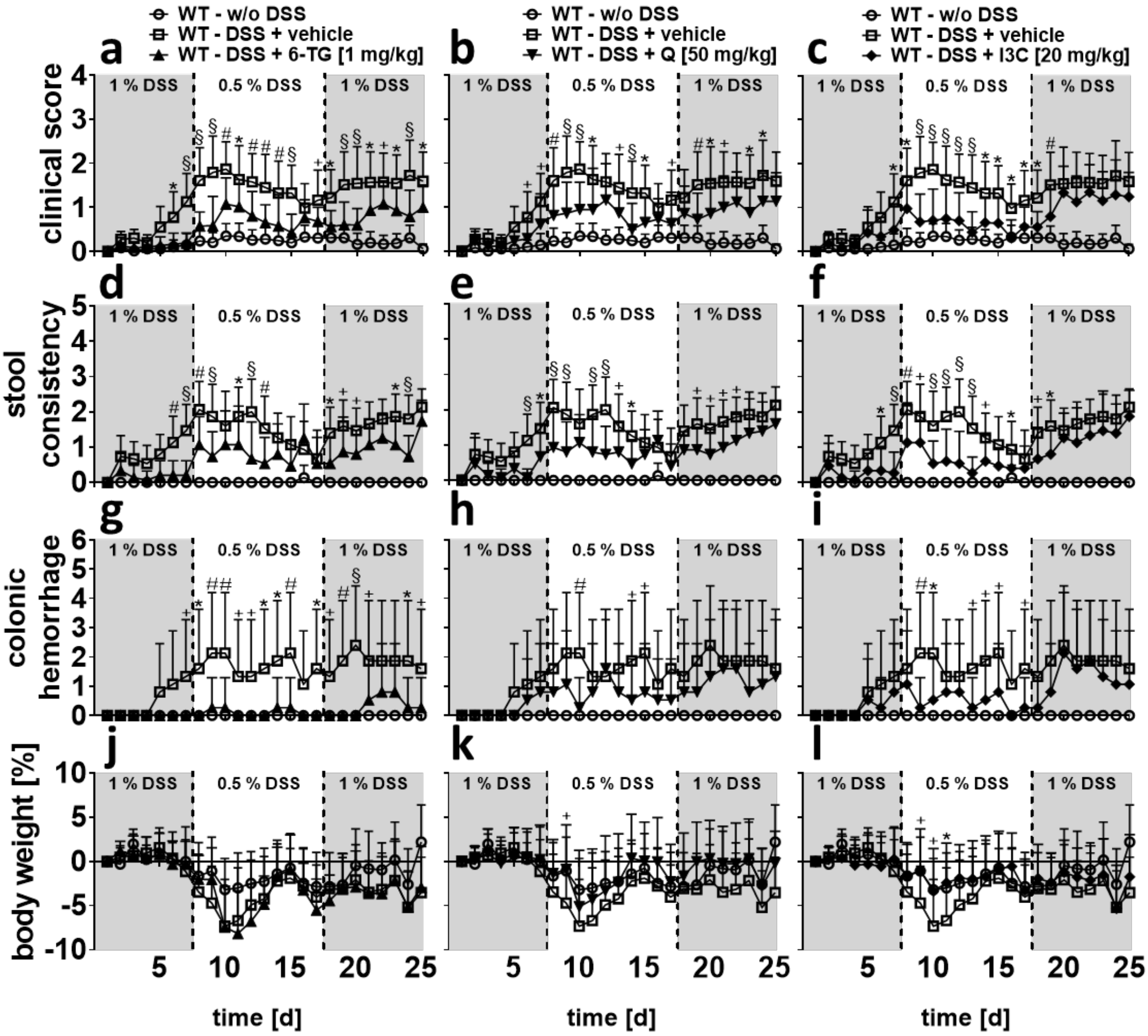

Figure S1. Therapeutic effects of Q and I3C during the course of chronic DSS colitis. Chronic colitis was induced in C57BL/6 wild-type (WT) mice by administering 1 % DSS in drinking water for 7 days, followed by 0.5 % DSS for 10 days, and again 1 % DSS for 7 days of. Animals were treated from days 1-10 and 18-22 with 6-thioguanine (6-TG; 1 mg/kg; **a**, **g**, **d**, **j**), quercetin (Q; 50 mg/kg; **b**, **e**, **h**, **k**) or indol-3-carbinol (I3C; 20 mg/kg; **c**, **f**, **i**, **l**) as indicated. Control animals were administered with PBS (1 % hydroxyethyl cellulose) by gavage (vehicle control). The clinical score (**a**, **b**, **c**) and its single parameters - stool consistency (**d**, **e**, **f**), colonic hemorrhage (**g**, **h**, **i**), and body weight loss (**j**, **k**, **l**) - were evaluated daily and are shown in the time course of the experiment as mean + SD. n=15 WT mice; +$P < 0.05$, *$P < 0.01$, #$P < 0.001$ and §$P < 0.0001$ vs the vehicle control group.

Table S1. Scoring system for stool consistency and blood in stool.

Scoring points	Stool consistency	Blood in stool
0	normal	no visible blood in stool
1	soft, but still formed	-
2	very soft	-
3	liquid	-
4	-	visible blood in stool

Table S2. Histological scoring system.

Finding	Grade	Definition
Fibrosis in submucosa	1	Focal lesion
	2	Several focal lesions
	3	Bridging lesion in the submucosa
	4	Like Grade 3, but with enlargement of submucosa
	5	Enlargement causes doubling and more of originally submucosa height
Fibrosis in mucosa	1	Focal lesion
	2	Several focal lesions
	3	Up to 50 % replaced
	4	Almost 80 % replaced
	5	Full replacement
Infiltration, granulocytes (normal: max. 2-3/hpf (x20))	1	10/hpf[1]
	2	20/hpf
	3	50/hpf
	4	Large, multifocal infiltration
	5	Infiltration forming compact masses of granulocytes
Infiltration, lymphocytes (normal: max. 2-3/hpf (x20))	1	10/hpf
	2	20/hpf
	3	50/hpf
	4	Large, multifocal infiltration
	5	Infiltration forming compact masses of lymphocytes
Infiltration, macrophages (normal: max. 2-3/hpf (x20))	1	10/hpf
	2	20/hpf
	3	50/hpf
	4	Large, multifocal infiltration
	5	Infiltration forming compact masses of macrophages
Edema	1	Very small lesions or focal only
	2	Several foci, may be confluenting
	3	Extending normal thickness of mucosa
	4	Extending normal thickness of intestine
	5	Associated with disruption of original structures
Necrosis	1	Very small lesions on mucosal surface
	2	Several foci, may be confluenting or one focus leading up to submucosa
	3	20-30 % of mucosa involved
	4	Up to 50 % of mucosa involved
	5	Complete mucosa involved

[1] hpf: high power field
